# Overcrowded Professions

**Published:** 1893-05

**Authors:** 


### Overcrowded Professions.

There is a general impression that we have too many doctors and lawyers; and the opinion has been rather freely expressed in various quarters that the trend of our public school system is to give the sons of farmers a sort of education which makes them discontented with their surroundings, and drives them to the larger centres. Among those thus influenced a large number go into the professions. The tendency of the age in this and other countries appears to be in the direction of centralization; and many give the public and high schools credit for creating a certain amount of such tendency.

It is, of course, rather an old story that too much education will injure certain classes. Few there are, however, that are likely to publicly advocate measures that will impair our grand public educational system. The majority do not care to go back to the ignorance of the dark ages, notwithstanding all the "bliss" that may be connected with that, sometimes, happy condition.

We are pleased to notice that the Government of Ontario appreciates the evils referred to, and is endeavoring to find a remedy. At the meeting of the Central Farmers' Institute, held in Toronto the second week in February, the Minister of Agriculture made the important announcement that an effort will be made in the public schools to so change the character of the teaching that the attention of the pupils will be directed towards "the beauties of rural life, the importance and dignity of labor, and the honor which may come to a man who excels in agricultural pursuits."

